# Supplementary material for: Clustering-independent estimation of cell abundances in bulk tissues using single-cell RNA-seq data
Source: Cell Rep Methods. 2024 Nov 18;4(11):100905. doi: 10.1016/j.crmeth.2024.100905 (PMC11705773; doi:10.1016/j.crmeth.2024.100905)
Supplement: Document S1. Figures S1–S7, Tables S1 and S2, and Methods S1 and S2 [file mmc1.pdf]

Cell Reports Methods, Volume *4*

Supplemental information

Clustering-independent estimation of cell abundances in bulk tissues using single-cell RNA-seq data

Rachael G. Aubin, Javier Montelongo, Robert Hu, Elijah Gunther, Patrick Nicodemus, and Pablo G. Camara

Supplemental Figures

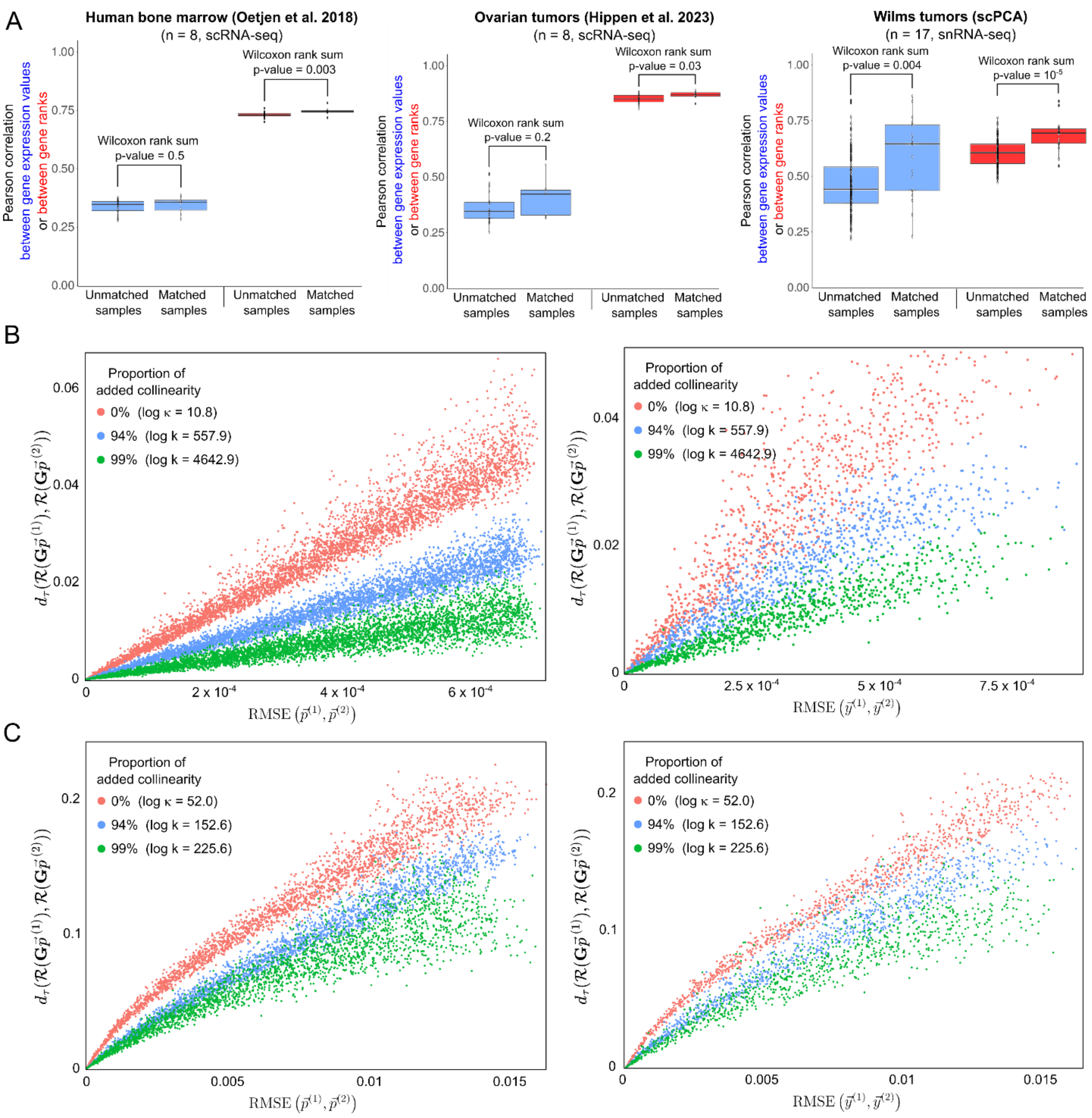

Figure S1. Using gene ranks to infer cell abundance from gene expression data of bulk tissues, related to STAR Methods. (A) Pearson correlation coefficient between the expression levels or between the gene ranks for 3 datasets consisting of single-cell/nucleus and bulk RNA-seq data from the same samples, encompassing human bone marrow [S1], high-grade serous ovarian tumors [S2], and Wilms tumors from the single-cell pediatric cancer atlas (scPCA). For

each sample, we aggregated the single cell or single nucleus counts across the cells to construct a synthetic bulk RNA-seq dataset for the sample. We then computed the Pearson correlation coefficient between the expression levels, or between the gene ranks, of the synthetic and actual bulk RNA-seq datasets using the 2,000 most variable genes. This analysis shows gene ranks from aggregated single-cell/nucleus can better discriminate between samples than aggregated gene expression values. In particular, the concordance between the ranks of the genes is higher than between the gene expression values, and the correlation between gene ranks in synthetic and real bulk datasets from the same individual ('matched samples') is significantly higher than in synthetic and real bulk datasets from different individuals ('unmatched samples'). **(B)** Distance (Kendall's τ distance, d_τ) in the space of gene rank correlation distributions as a function of the distance in the space of cell abundance distributions (root mean square error, RMSE) for various levels of collinearity in the gene expression matrix $\boldsymbol{G}$. Each point corresponds to a pair of simulated random cell abundance distributions over 8,000 cells from a human bone-marrow single-cell RNA-seq dataset[1]. The bulk gene expression profile corresponding to each simulated distribution is obtained by aggregating the single-cell gene expression counts of the individual cells (left) or top 10 principal components (right) according to their probability for the 2,000 most variable genes. Additional collinearity is included by replacing the gene expression profile of a fraction of the cells with rescaled copies of the expression profile of other cells in the single-cell dataset. The amount of added collinearity and the logarithm of the resulting condition number for the gene expression matrix ($\log \kappa$) are indicated. As predicted from the mathematical foundation of ConDecon, for a sufficiently large number of variable genes, the distance between two bulk datasets in the space of rank correlations is small ($d_\tau\left(\mathcal{R}\left(\boldsymbol{G}\vec{p}^{(1)}\right), \mathcal{R}\left(\boldsymbol{G}\vec{p}^{(2)}\right)\right) \simeq 0$) if and only if their cell abundance composition is very similar ($\mathrm{RMSE}\left(\vec{p}^{(1)}, \vec{p}^{(2)}\right) \simeq 0$). **(C)** Same as in (B), but the simulated random cell abundance distributions are built by sampling from the probability simplex using beta distributions $\beta(a_1, a_2)$ and $\beta(a_2, a_1)$, with $a_1 = 0.5$ and $a_2 = 8$, instead of uniform sampling.

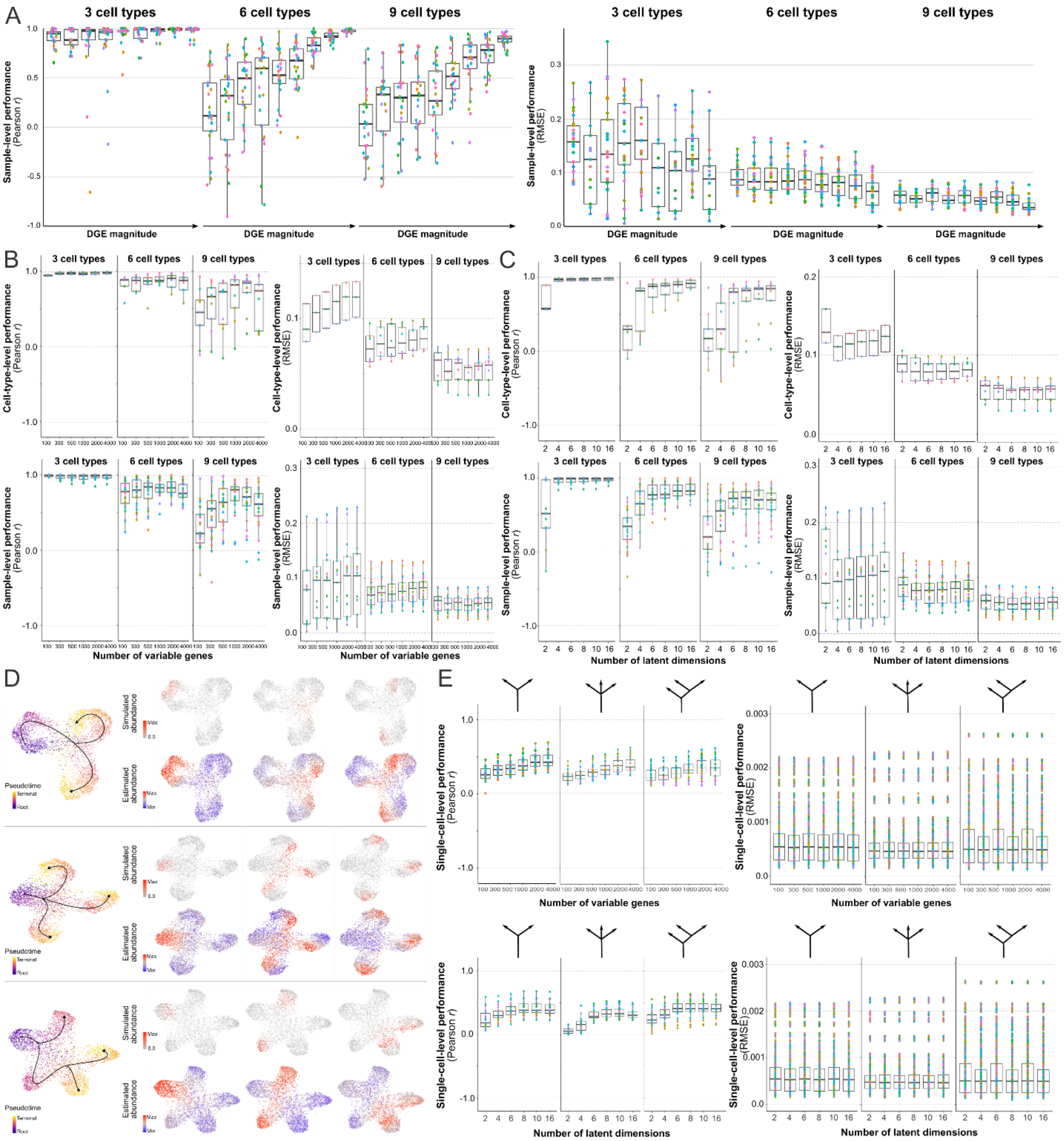

Figure S2. Deconvolution of simulated bulk RNA-seq data of discrete cell populations and continuous cell differentiation processes, related to Figure 2. (A) Sample-level Pearson correlation coefficient and root mean square error (RMSE) between simulated and estimated cell population abundances in simulations of bulk RNA-seq datasets (*n* = 675) with 3, 6, or 9 discrete

cell populations and varying degree of differential gene expression (DGE). **(B, C)** Cell-type- and sample-level Pearson correlation coefficient and RMSE between simulated and estimated cell population abundances in simulated bulk RNA-seq datasets (n = 675) with 3, 6, or 9 discrete cell populations as a function of the number of variable genes (B) and latent dimensions (C) used by ConDecon. **(D)** Cell abundance estimation in 9 simulated bulk RNA-seq datasets of 3 cell differentiation processes with 1 precursor and 2 or 3 terminally differentiated cell states. Left: The UMAP representation of each simulated single-cell RNA-seq dataset is colored by the simulated pseudotime. Right: For each single-cell dataset, the simulated (top) and estimated (bottom) cell abundances are shown for 3 bulk RNA-seq datasets constructed by sampling cells non-uniformly from the single-cell dataset. **(E)** Single-cell-level Pearson correlation coefficient and RMSE between simulated and estimated cell abundances in simulations of bulk RNA-seq datasets with 3 different topologies as a function of the number of variable genes (top) and latent dimensions (bottom) used by ConDecon. The topologies of the cell differentiation processes are indicated at the top.

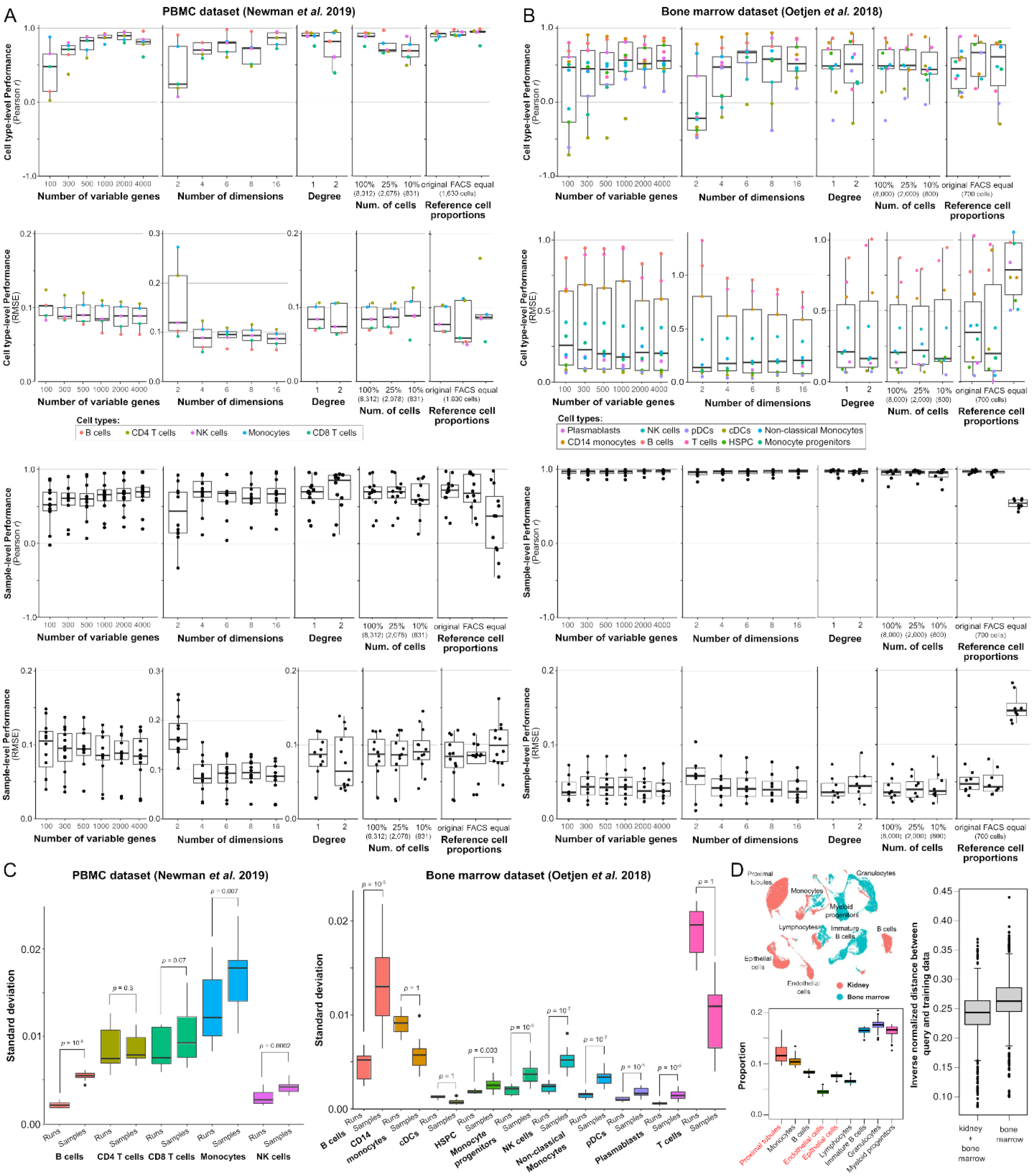

Figure S3. Deconvolution of bulk RNA-seq data of PBMCs and bone marrow as a function of ConDecon's parameters, related to Figure 3. (A, B) Single-cell-level Pearson correlation coefficient and root mean square error (RMSE) between the estimated cell abundances by ConDecon and the observed abundances by FACS across samples (cell-type-level performance)

and cell types (sample-level performance) for bulk RNA-seq datasets consisting of 8 bone marrow [S1] (A) and 12 PBMC [S3] (B) samples as a function of the number of variable genes, the number of latent dimensions, the degree of the polynomial, the number of cells in the reference single-cell RNA-seq dataset, and the similitude between the cell proportions in the reference single-cell data set and the query bulk data, where "original", "FACS", and "equal" indicate the proportions in the original single-cell dataset, in the FACS data, or equal proportions for all the cell populations present in the reference single-cell dataset, respectively. **(C)** Standard deviation of the cell type abundance inferences of ConDecon across 20 different random initializations ("runs") and across samples ("samples"). The variability of the inferred abundances across runs is significantly smaller than the variability across samples for almost all cell types. 1-sided Wilcoxon rank sum test *p*-values are indicated. **(D)** Deconvolution of mouse bone marrow bulk data using reference single-cell RNA-seq data of mouse bone marrow and kidney from the Tabula Muris Senis [S4]. Left, top: UMAP representation of the combined kidney and bone marrow single-cell RNA-seq datasets. Left, bottom: Inferred cell type abundances for bulk RNA-seq data from 53 bone marrow samples. The inferences of ConDecon are affected by the large mismatch between the reference and query datasets, with 24% of the probability mass assigned to kidney-specific cell populations (indicated in red). Right: The inverse distance between the point that corresponds to the query bulk sample and the 10 nearest training data points in the space of probability distributions, normalized by the average distance between training data points, can be used as an indicator of the quality of the inferences made by ConDecon. The inverse distance varies between 0 (for single-cell reference data unrelated to the query bulk data) and approximately 1 (for single-cell reference data that accurately match the query bulk data). In the figure, the inverse distance is significantly increased when using a bone marrow instead of a combined bone marrow and kidney single-cell RNA-seq dataset to deconvolve the bone marrow bulk RNA-seq data (2-sided Wilcoxon rank-sum test *p*-value $< 10^{-16}$).

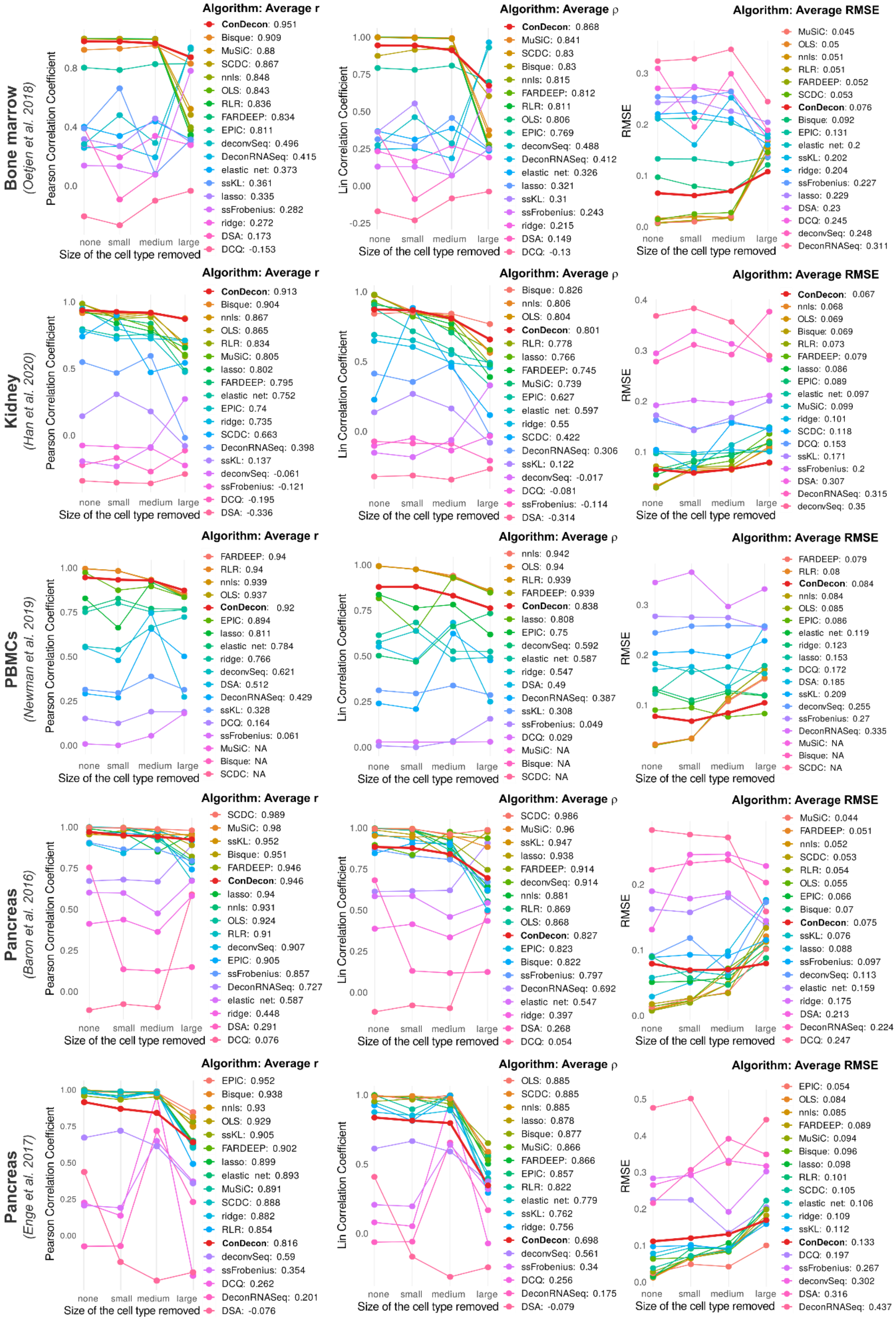
Bone marrow
(Oetjen et al. 2018)
Pearson Correlation Coefficient
Size of the cell type removed
none small medium large
Algorithm: Average r
ConDecon: 0.951
Bisque: 0.909
MuSiC: 0.88
SCDC: 0.867
nnls: 0.848
OLS: 0.843
RLR: 0.836
FARDEEP: 0.834
EPIC: 0.811
deconvSeq: 0.496
DeconRNASeq: 0.415
elastic net: 0.373
ssKL: 0.361
lasso: 0.335
ssFrobenius: 0.282
ridge: 0.272
DSA: 0.173
DCQ: -0.153
Lin Correlation Coefficient
Algorithm: Average ρ
ConDecon: 0.868
MuSiC: 0.841
SCDC: 0.83
Bisque: 0.83
nnls: 0.815
FARDEEP: 0.812
RLR: 0.811
OLS: 0.806
EPIC: 0.769
deconvSeq: 0.488
DeconRNASeq: 0.412
elastic net: 0.326
lasso: 0.321
ssKL: 0.31
ssFrobenius: 0.243
ridge: 0.215
DSA: 0.149
DCQ: -0.13
RMSE
Algorithm: Average RMSE
MuSiC: 0.045
OLS: 0.05
nnls: 0.051
RLR: 0.051
FARDEEP: 0.052
SCDC: 0.053
ConDecon: 0.076
Bisque: 0.092
EPIC: 0.131
elastic net: 0.2
ssKL: 0.202
ridge: 0.204
ssFrobenius: 0.227
lasso: 0.229
DSA: 0.23
DCQ: 0.245
deconvSeq: 0.248
DeconRNASeq: 0.311
Kidney
(Han et al. 2020)
Algorithm: Average r
ConDecon: 0.913
Bisque: 0.904
nnls: 0.867
OLS: 0.865
RLR: 0.834
MuSiC: 0.805
lasso: 0.802
FARDEEP: 0.795
elastic net: 0.752
EPIC: 0.74
ridge: 0.735
SCDC: 0.663
DeconRNASeq: 0.398
ssKL: 0.137
deconvSeq: -0.061
ssFrobenius: -0.121
DCQ: -0.195
DSA: -0.336
Algorithm: Average ρ
Bisque: 0.826
nnls: 0.806
OLS: 0.804
ConDecon: 0.801
RLR: 0.778
lasso: 0.766
FARDEEP: 0.745
MuSiC: 0.739
EPIC: 0.627
elastic net: 0.597
ridge: 0.55
SCDC: 0.422
DeconRNASeq: 0.306
ssKL: 0.122
deconvSeq: -0.017
DCQ: -0.081
ssFrobenius: -0.114
DSA: -0.314
Algorithm: Average RMSE
ConDecon: 0.067
nnls: 0.068
OLS: 0.069
Bisque: 0.069
RLR: 0.073
FARDEEP: 0.079
lasso: 0.086
EPIC: 0.089
elastic net: 0.097
MuSiC: 0.099
ridge: 0.101
SCDC: 0.118
DCQ: 0.153
ssKL: 0.171
ssFrobenius: 0.2
DSA: 0.307
DeconRNASeq: 0.315
deconvSeq: 0.35
PBMCs
(Newman et al. 2019)
Algorithm: Average r
FARDEEP: 0.94
RLR: 0.94
nnls: 0.939
OLS: 0.937
ConDecon: 0.92
EPIC: 0.894
lasso: 0.811
elastic net: 0.784
ridge: 0.766
deconvSeq: 0.621
DSA: 0.512
DeconRNASeq: 0.429
ssKL: 0.328
DCQ: 0.164
ssFrobenius: 0.061
MuSiC: NA
Bisque: NA
SCDC: NA
Algorithm: Average ρ
nnls: 0.942
OLS: 0.94
RLR: 0.939
FARDEEP: 0.939
ConDecon: 0.838
lasso: 0.808
EPIC: 0.75
deconvSeq: 0.592
elastic net: 0.587
ridge: 0.547
DSA: 0.49
DeconRNASeq: 0.387
ssKL: 0.308
ssFrobenius: 0.049
DCQ: 0.029
MuSiC: NA
Bisque: NA
SCDC: NA
Algorithm: Average RMSE
FARDEEP: 0.079
RLR: 0.08
ConDecon: 0.084
nnls: 0.084
OLS: 0.085
EPIC: 0.086
elastic net: 0.119
ridge: 0.123
lasso: 0.153
DCQ: 0.172
DSA: 0.185
ssKL: 0.209
deconvSeq: 0.255
ssFrobenius: 0.27
DeconRNASeq: 0.335
MuSiC: NA
Bisque: NA
SCDC: NA
Pancreas
(Baron et al. 2016)
Algorithm: Average r
SCDC: 0.989
MuSiC: 0.98
ssKL: 0.952
Bisque: 0.951
FARDEEP: 0.946
ConDecon: 0.946
lasso: 0.94
nnls: 0.931
OLS: 0.924
RLR: 0.91
deconvSeq: 0.907
EPIC: 0.905
ssFrobenius: 0.857
DeconRNASeq: 0.727
elastic net: 0.587
ridge: 0.448
DSA: 0.291
DCQ: 0.076
Algorithm: Average ρ
SCDC: 0.986
MuSiC: 0.96
ssKL: 0.947
lasso: 0.938
FARDEEP: 0.914
deconvSeq: 0.914
nnls: 0.881
RLR: 0.869
OLS: 0.868
ConDecon: 0.827
EPIC: 0.823
Bisque: 0.822
ssFrobenius: 0.797
DeconRNASeq: 0.692
elastic net: 0.547
ridge: 0.397
DSA: 0.268
DCQ: 0.054
Algorithm: Average RMSE
MuSiC: 0.044
FARDEEP: 0.051
nnls: 0.052
SCDC: 0.053
RLR: 0.054
OLS: 0.055
EPIC: 0.066
Bisque: 0.07
ConDecon: 0.075
ssKL: 0.076
lasso: 0.088
ssFrobenius: 0.097
deconvSeq: 0.113
elastic net: 0.159
ridge: 0.175
DSA: 0.213
DeconRNASeq: 0.224
DCQ: 0.247
Pancreas
(Enge et al. 2017)
Algorithm: Average r
EPIC: 0.952
Bisque: 0.938
nnls: 0.93
OLS: 0.929
ssKL: 0.905
FARDEEP: 0.902
lasso: 0.899
elastic net: 0.893
MuSiC: 0.891
SCDC: 0.888
ridge: 0.882
RLR: 0.854
ConDecon: 0.816
deconvSeq: 0.59
ssFrobenius: 0.354
DCQ: 0.262
DeconRNASeq: 0.201
DSA: -0.076
Algorithm: Average ρ
OLS: 0.885
SCDC: 0.885
nnls: 0.885
lasso: 0.878
Bisque: 0.877
MuSiC: 0.866
FARDEEP: 0.866
EPIC: 0.857
RLR: 0.822
elastic net: 0.779
ssKL: 0.762
ridge: 0.756
ConDecon: 0.698
deconvSeq: 0.561
ssFrobenius: 0.34
DCQ: 0.256
DeconRNASeq: 0.175
DSA: -0.079
Algorithm: Average RMSE
EPIC: 0.054
OLS: 0.084
nnls: 0.085
FARDEEP: 0.089
MuSiC: 0.094
Bisque: 0.096
lasso: 0.098
RLR: 0.101
SCDC: 0.105
elastic net: 0.106
ridge: 0.109
ssKL: 0.112
ConDecon: 0.133
DCQ: 0.197
ssFrobenius: 0.267
deconvSeq: 0.302
DSA: 0.316
DeconRNASeq: 0.437

Figure S4. Benchmarking of the aggregated cell abundance estimates of ConDecon in comparison to seventeen other methods for gene expression deconvolution, related to Figure 3. Evaluation of the aggregated cell abundance estimates of ConDecon and the cell type abundance estimates of 17 other deconvolution methods across 5 datasets using the benchmarking pipeline of Avila-Cobos *et al.* For each algorithm and dataset, the Pearson's correlation coefficient (left), the Lin's concordance correlation coefficient, and the root mean squared error (RMSE) (right) of the estimates, combined across samples and cell types, is shown for cases where there is none, one small, one medium, or one large cell population missing in the reference single-cell data.

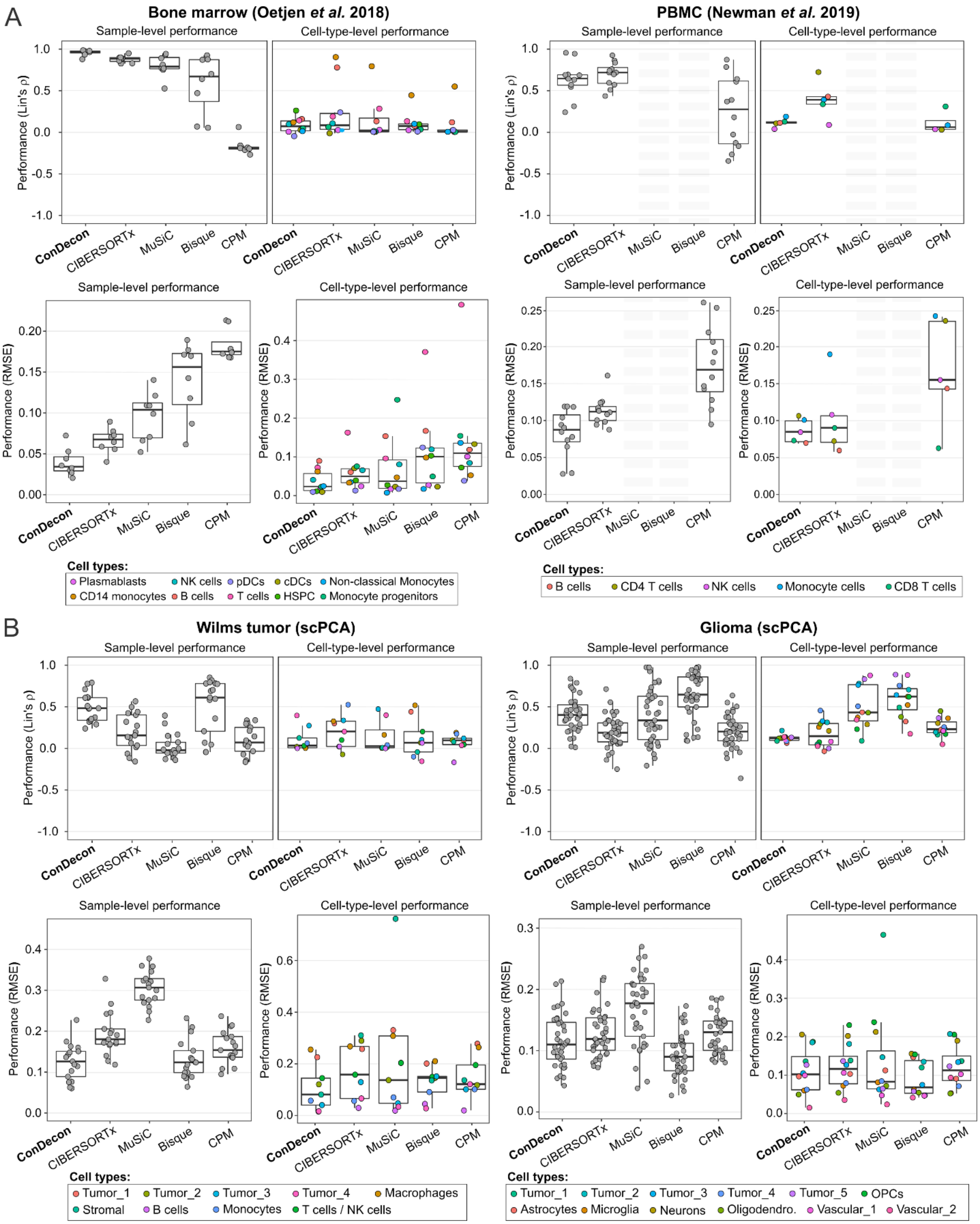

Figure S5. Comparison between cell type abundance estimates derived from FACS and single-nucleus RNA-seq data and those from ConDecon and 4 other deconvolution

methods, related to Figure 3. (A, B) Two bulk RNA-seq datasets consisting of 8 bone marrow [S1] (A, left) and 12 PBMC [S3] (A, right) samples, for which paired FACS data are available, as well as two bulk RNA-seq datasets consisting of 17 Wilms tumor (B, right) and 37 pediatric glioma (B, left) samples, for which paired single-nuclei RNA-seq data are available, were considered. The sample-level and cell-type-level RMSE and Lin's concordance correlation coefficient are shown for each algorithm in each dataset. We were unable to apply MuSiC and Bisque to the PBMC dataset since these methods require that the reference single-cell RNA-seq data consists of at least 2 biological replicates.

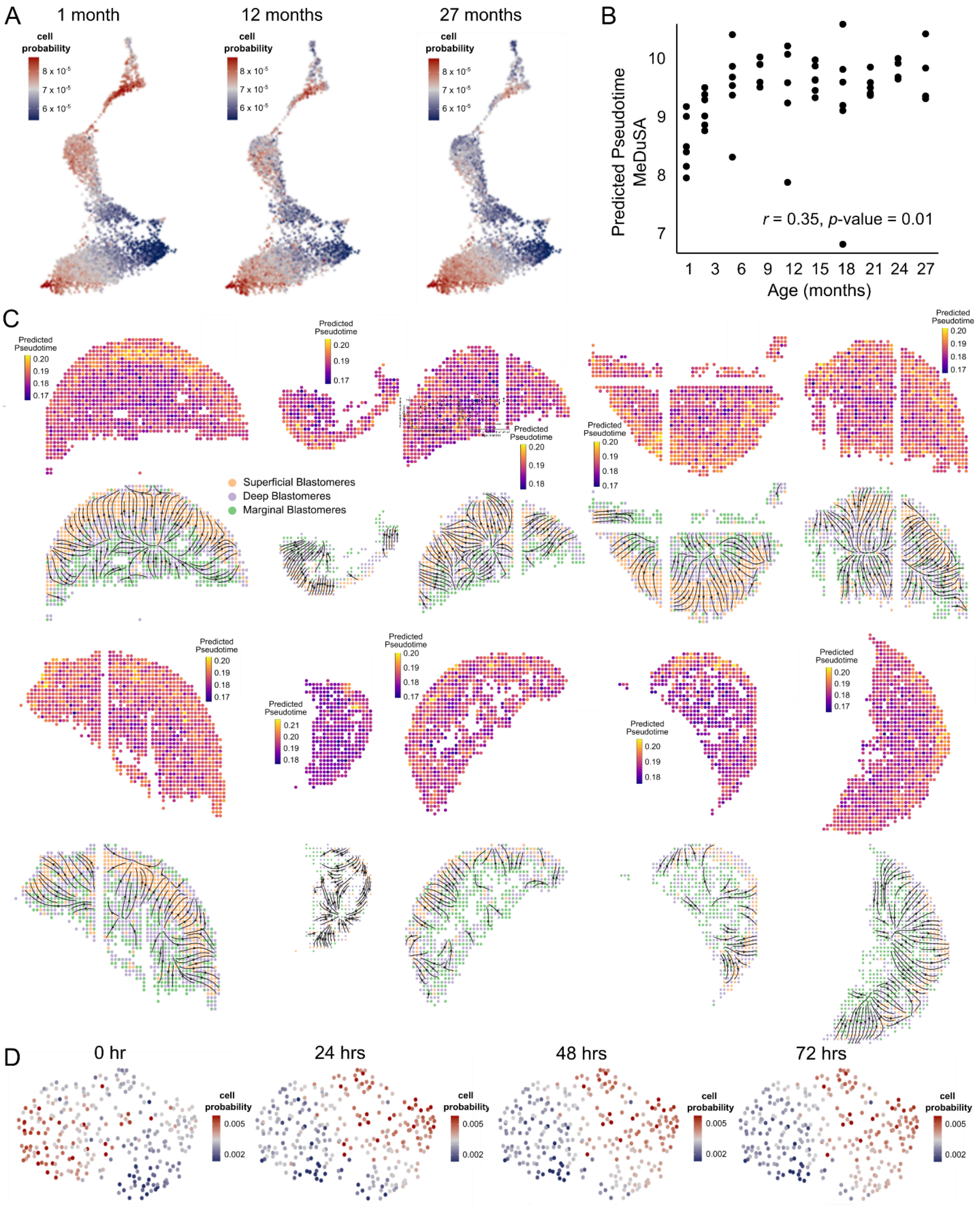

Figure S6. Deconvolution of continuous cellular processes, related to Figures 4 and 5.

(A) Identification of age-associated changes in B-cell maturation using bulk bone marrow

tissues. Single-cell abundances inferred by ConDecon for three bone marrow samples from 1-, 12-, and 27-months old mice profiled with bulk RNA-seq by the Tabula Muris Consortium [S4]. **(B)** Average pseudotime inferred by MeDuSA for the B cells in each bulk sample as a function of the mice age, for bone marrow samples of 53 mice profiled with bulk RNA-seq. Pearson's correlation coefficient r = 0.35, p-value = 0.01. **(C)** Deconvolution of spatial transcriptomic data of zebrafish embryos. Spatial tissue sections of 10 3.3 hpf zebrafish embryos profiled with Stereo-seq [S5]. Each section is labeled by the average pseudotime estimated with ConDecon for the cells in each pixel (top) and the corresponding spatial cell differentiation trajectories (bottom). **(D)** UMAP representation of the single-cell ATAC-seq data of a patient-derived melanoma cell line (MM087) profiled 0, 24, 48, and 72 hours after knocking out SOX10 [S6]. The representation is colored by the single-cell abundances estimated with ConDecon for 4 samples from a different melanoma cell line (MM057) profiled with bulk ATAC-seq 0, 24, 48, and 72 hours after knocking out SOX10.

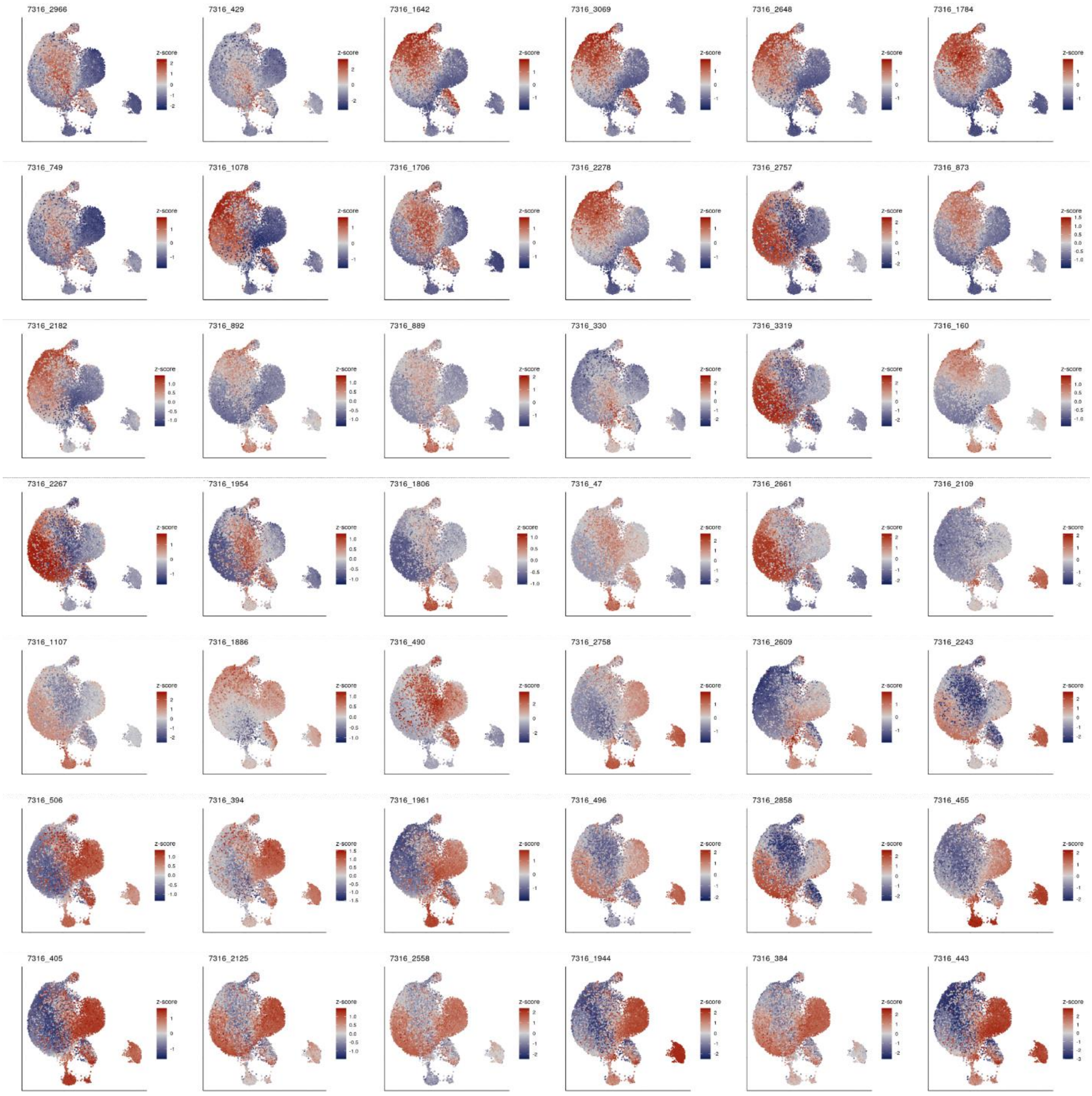

Figure S7. Deconvolution of bulk RNA-seq data of pediatric ependymoma, related to Figure 6. Single-cell abundance estimates computed with ConDecon for 42 pediatric ependymal tumors in the posterior fossa profiled with bulk RNA-seq. The UMAP representation of 25,349 cells from 9 posterior fossa ependymal tumors profiled with single-nucleus RNA-seq in Aubin *et al.* [S7] is colored by the inferred single-cell abundances. Tumors are arranged from

left to right and top to bottom according to their inferred state in the neuroepithelial-to-mesenchymal transition.

Methods S1: Using gene ranks to infer cell abundances, related to Figure 1 and STAR Methods.

Given the gene expression profile of a bulk tissue, our goal is to infer the point in the space of cell abundance distributions over a reference single-cell RNA-seq dataset (of the same tissue type but possibly involving different cell abundances) that most closely represents the query bulk tissue. To reduce the effect of technical differences between single-cell RNA-seq and bulk RNA-seq measurements, we utilize gene ranks to compare the gene expression profile that results from aggregating single-cell gene expression levels across cells with the gene expression profile of the bulk tissue (Figure S1A). While different cell abundance configurations can lead to the same vector of gene ranks, this concern can be safely disregarded when working with single-cell datasets consisting of hundreds to thousands of variable genes.

Consider a reference single-cell dataset consisting of J cells and T variable genes and let $\boldsymbol{G}$ be the $T \times J$ expression table. The space of possible relative cell abundances in a synthetic bulk tissue constructed by sampling cells from the reference single-cell dataset consists of a $(J-1)$-simplex, since cell proportions must add to 1. For example, in the case of 3 cells, the space of cell abundances consists of a triangle with unit-length sides, as illustrated below. For each point in the space of cell abundances, we can form a synthetic bulk RNA-seq dataset by aggregating the columns of $\boldsymbol{G}$ using weights given by the relative cell abundance of each cell. A bulk RNA-seq dataset can then be represented as a point in a J-dimensional space of gene rank correlations, where each dimension represents the value of the gene rank correlation distance of the bulk dataset with a cell in the reference single-cell dataset. Multiple points in the space of cell abundances may lead to the same point in the space of rank correlations, leading to a tessellation of the space of cell abundances. However, since the map between the space cell abundances and the space of gene rank correlations preserves local neighborhoods, we can think of the points in the space of gene rank correlations as a non-uniform pixelation of the space of cell abundances,

where the resolution of the pixelation is controlled by the number of variable genes. Thus, for a sufficiently large number of variable genes (see Methods), it is possible to infer the relative cell abundances associated with a bulk RNA-seq dataset with high accuracy based on the gene rank correlations with the cells in a reference single-cell dataset, as shown below for a toy example consisting of 3 cells. The algorithm ConDecon tries to learn the map between the space of cell abundances and the space of gene rank correlations to infer cell abundances from bulk datasets.

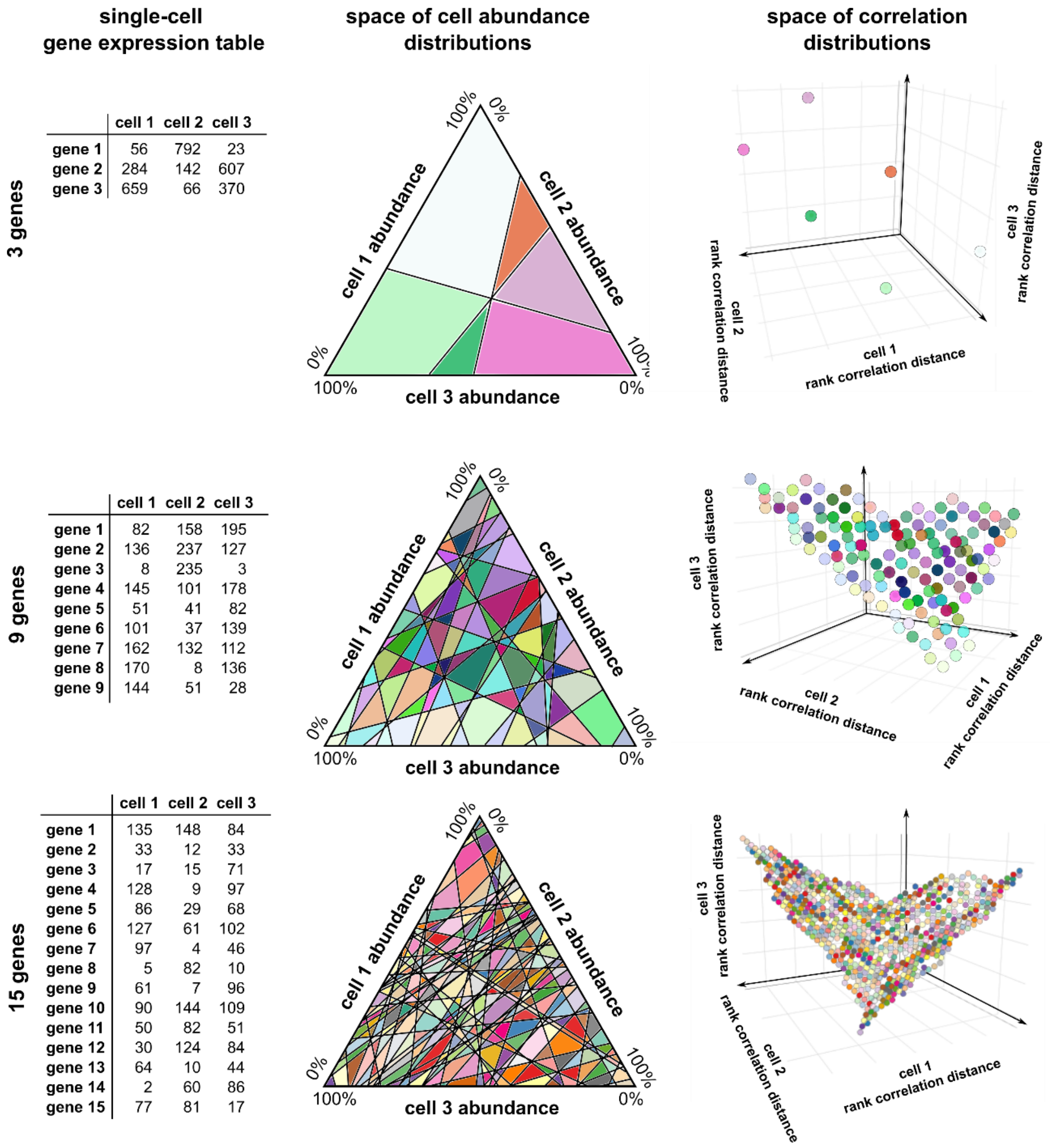

	cell 1	cell 2	cell 3
gene 1	56	792	23
gene 2	284	142	607
gene 3	659	66	370

	cell 1	cell 2	cell 3
gene 1	82	158	195
gene 2	136	237	127
gene 3	8	235	3
gene 4	145	101	178
gene 5	51	41	82
gene 6	101	37	139
gene 7	162	132	112
gene 8	170	8	136
gene 9	144	51	28

	cell 1	cell 2	cell 3
gene 1	135	148	84
gene 2	33	12	33
gene 3	17	15	71
gene 4	128	9	97
gene 5	86	29	68
gene 6	127	61	102
gene 7	97	4	46
gene 8	5	82	10
gene 9	61	7	96
gene 10	90	144	109
gene 11	50	82	51
gene 12	30	124	84
gene 13	64	10	44
gene 14	2	60	86
gene 15	77	81	17

Methods S2: Detailed Protocol, related to Figures 1, 2, 4, and 5.

The aim of ConDecon is to infer a probability distribution across a reference single-cell RNA-seq dataset that represents the likelihood for each cell in the reference data to be present in the query bulk tissue. To that end, ConDecon requires three inputs:

- Single-cell gene expression count data.
- Single-cell latent space.
- Normalized bulk data.

With this information, ConDecon learns a relationship that explains the similarity between the gene expression profile of bulk and single-cell data as a function of changes in cell abundances, without relying on cluster labels or cell-type specific gene expression signatures at any step. In this Detailed Protocol, we demonstrate the installation of ConDecon and its application in several examples. A more complete guide of ConDecon and its detailed documentation can be found in https://camaralab.github.io/ConDecon/index.html.

Installation

ConDecon requires an installation of R version 2.10 or later, including the package `devtools`. We recommend using RStudio notebooks (https://posit.co/download/rstudio-desktop/) to run ConDecon, as they provide a nice interactive environment for data analysis.

To install and load ConDecon, run the commands:

```
devtools::install_github("CamaraLab/ConDecon")
library(ConDecon)
```

Quick example with simulated data

In this first example, we will apply ConDecon to simulated transcriptomic data and visualize the expected results. We first load the necessary packages,

```r
library(ConDecon)
library(ggplot2)
```

As a reference dataset, we will use simulated single-cell RNA-seq data containing 9 clusters/groups (gps). This data was generated using the software Splatter [S8]. We will start by loading the single cell count table and metadata provided by the ConDecon package.

```r
# Single-cell gene expression count data
data(counts_gps)

# Single-cell PCA latent space
data(latent_gps)

# Top 2,000 variable genes
data(variable_genes_gps)

# Meta data of single-cell RNA-seq data
data(meta_data_gps)
```

```r
# Visualize the cluster annotations of the single-cell RNA seq data
ggplot(data.frame(meta_data_gps), aes(x = UMAP_1, y = UMAP_2, color = celltypes)) +
  geom_point(size = 1.5) +
  theme_classic()
```

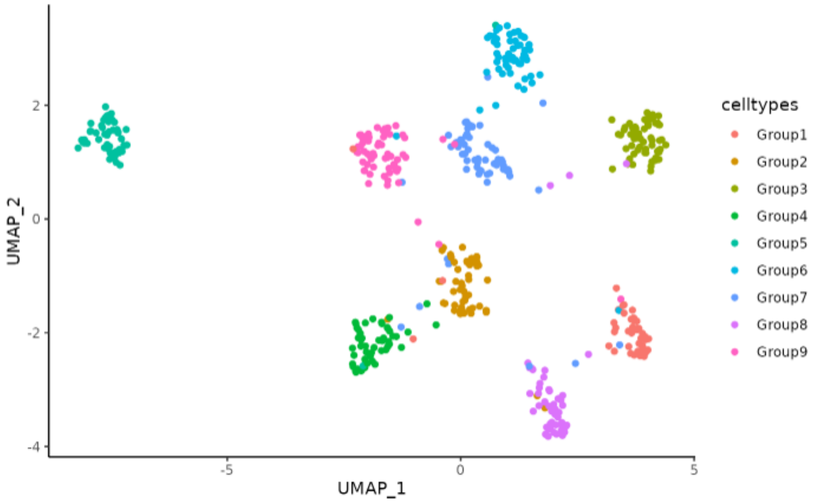

We will use ConDecon to deconvolve 5 simulated bulk transcriptomic profiles.

```
# Bulk gene expression data, normalized by TPMs
data("bulk_gps")
```

`RunConDecon()` is the main function necessary to infer cell abundances for each input bulk sample. This function requires 4 inputs:

1. Single cell count matrix.
2. Single-cell latent space matrix.
3. Character vector of variable features associated with the single-cell data.
4. Normalized bulk data matrix.

The output of this function is a ConDecon object containing a `Normalized_cell.probs` matrix with the predicted cell probability distributions.

```
ConDecon_obj = RunConDecon(counts = counts_gps,
                           latent = latent_gps,
                           variable.features = variable_genes_gps,
                           bulk = bulk_gps,
                           dims = 10)
#> Warning in pdist::pdist(t(cond$TrainingSet$bulk_coefficients),
#> t(cond$TrainingSet$bulk_coefficients)): Y is the same as X, did you mean to use
#> dist instead?
```

With `PlotConDecon()`, we can visualize the relative cell probabilities of each of the 5 bulk samples.

```
PlotConDecon(ConDecon_obj = ConDecon_obj,
             umap = meta_data_gps[,c("UMAP_1", "UMAP_2")])
```

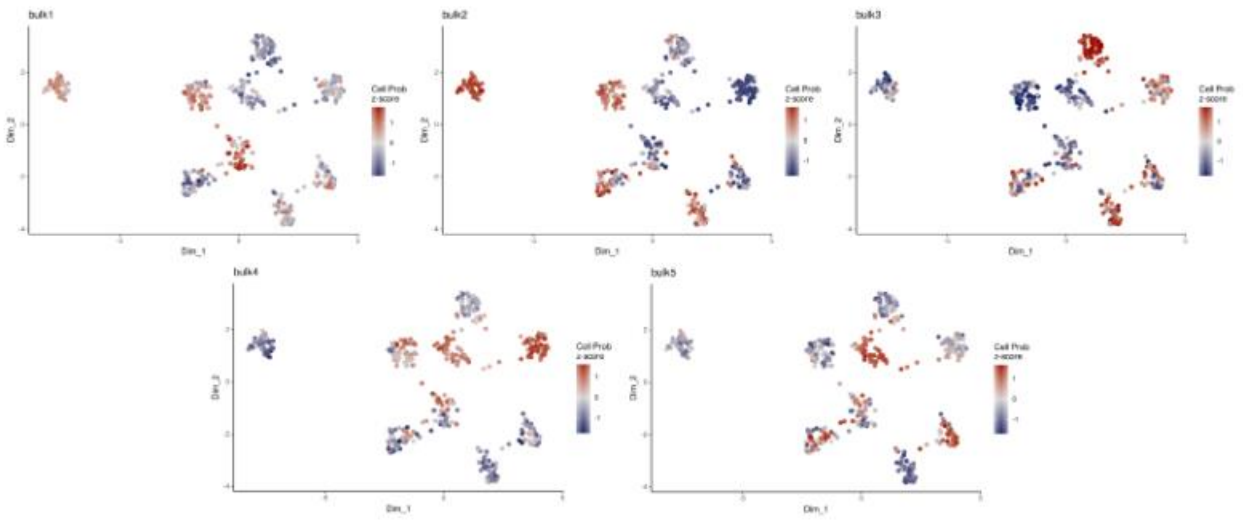

To visualize the actual cell probabilities in each sample we can set `relative=F` in the above command:

```
PlotConDecon(ConDecon_obj = ConDecon_obj,
             umap = meta_data_gps[,c("UMAP_1", "UMAP_2")], relative = F)
```

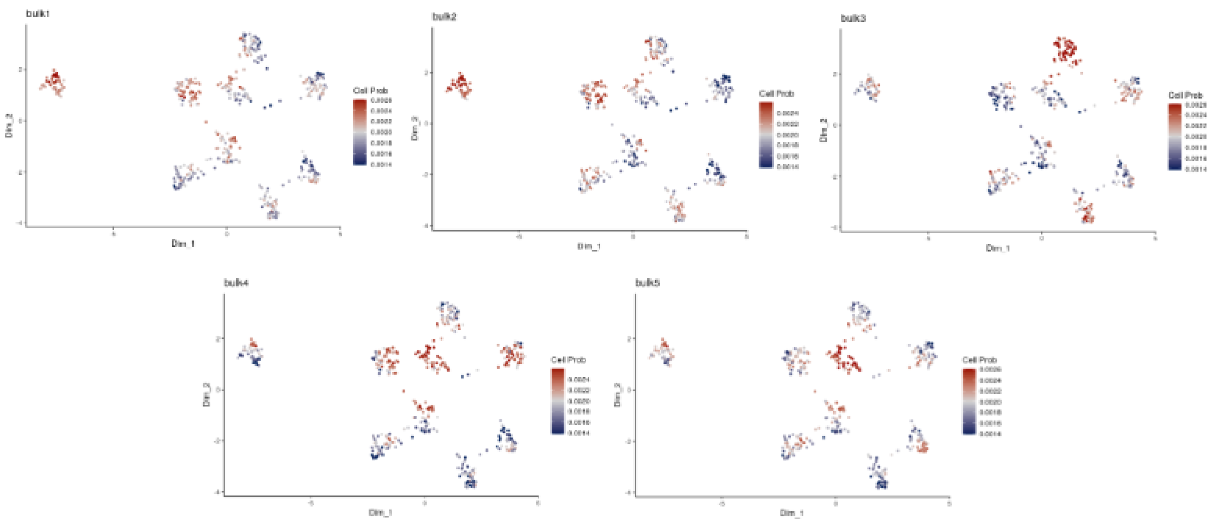

Finally, we can compare ConDecon's predictions to the true cell type proportions of each simulated bulk sample.

```
data(true_prop_gps)

for(i in 1:5){
  plot(ggplot(data=true_prop_gps, aes_string(x="celltypes", y=paste0("bulk", i),
                                              fill = "celltypes")) +
    geom_bar(stat="identity") +
    theme_classic())
}
```

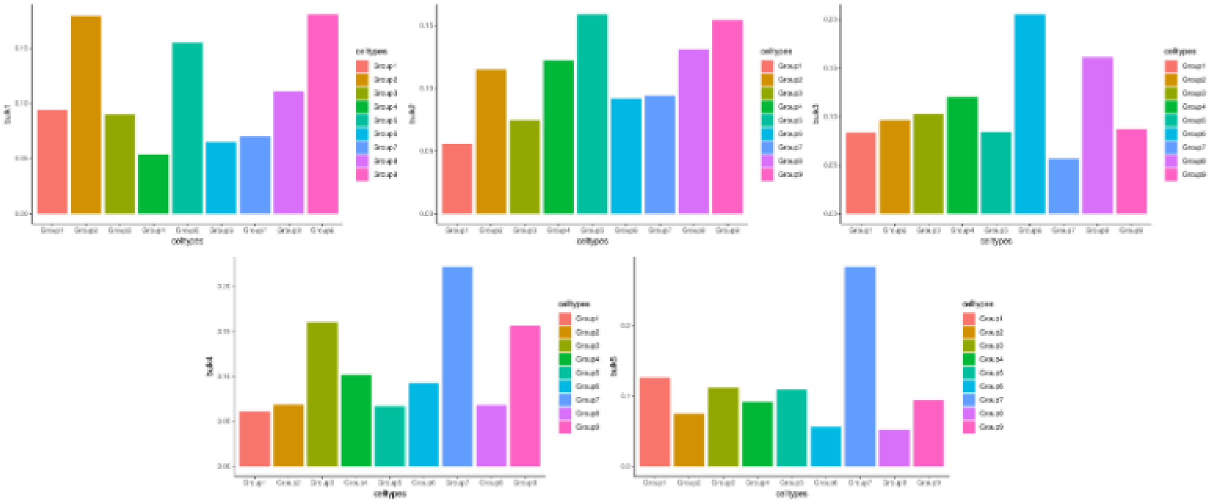

Deconvolution of bulk RNA-seq data

In this example, we will use ConDecon to study changes in single-cell abundance associated with continuous cellular processes. We will consider single-cell and bulk RNA-seq data from the bone marrow of mice ages 1 – 27 months profiled by the Tabula Muris Consortium [S4] and use these data to study changes in cell abundance associated with development and aging. We are particularly interested in well-characterized changes in B-cell abundance that occur during postnatal development. For convenience, the processed data associated with this tutorial is available on Zenodo (https://zenodo.org/record/7604026#.Y-EzEC_MIuU).

```
library(ConDecon)
library(ggplot2)
library(gridExtra)
```

As a reference dataset, we will use single-cell RNA-seq data from across the life span of mice.

```
scRNA_count <- readRDS("../scRNA_counts.Rds")
scRNA_Harmony <- readRDS("../scRNA_Harmony.Rds")
scRNA_var_genes <- readRDS("../scRNA_var_genes.Rds")
scRNA_meta_data <- readRDS("../scRNA_meta_data.Rds")
```

```
# Visualize the cell types of the single-cell RNA-seq data
g1 <- ggplot(scRNA_meta_data, aes(x = UMAP_1, y = UMAP_2, color = cellType)) +
  geom_point(size = 0.5) +
  theme_classic()
# Visualize the mouse age of the single-cell RNA-seq data
g2 <- ggplot(scRNA_meta_data, aes(x = UMAP_1, y = UMAP_2,
                           color = factor(as.character(Age_months), levels = c(3,18,24)))) +
  geom_point(size = 0.5) +
  theme_classic()

grid.arrange(g1, g2, ncol=2)
```

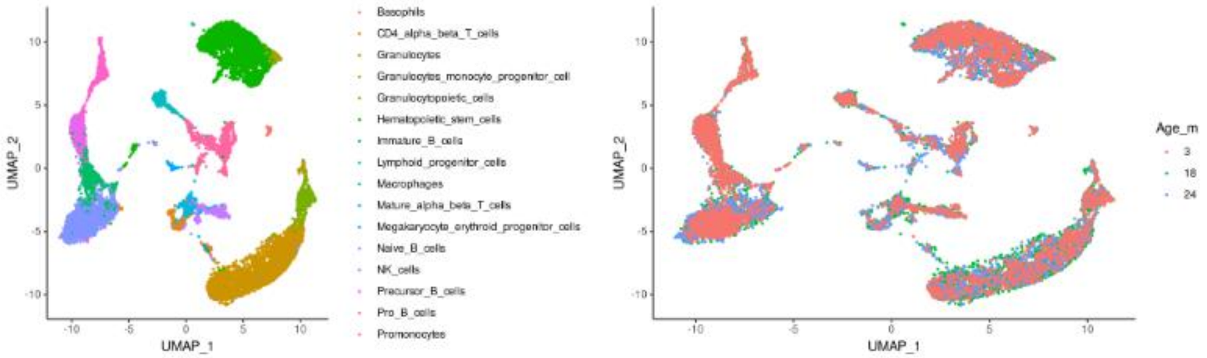

We are interested in using ConDecon to deconvolve 53 bulk RNA-seq samples from 10 time points.

```
BulkRNA_data <- readRDS("../BulkRNA_data.Rds")
BulkRNA_meta_data <- readRDS("../BulkRNA_meta_data.Rds")
```

We can now use `RunConDecon()` to deconvolve bulk RNA-seq data using single-cell RNA-seq data as reference.

```r
startTime <- Sys.time()

ConDecon_obj = RunConDecon(counts = scRNA_count,
                           latent = scRNA_Harmony[,1:30],
                           bulk = BulkRNA_data,
                           variable.features = scRNA_var_genes)

endTime <- Sys.time()
print(endTime - startTime)
#Time difference of 17.17469 mins
```

With `PlotConDecon()`, we can visualize the relative cell probabilities of each bulk sample. We find that ConDecon can recapitulate the continuous transition from an abundance of pro B-cells in young mice (≤ 3 months) to an abundance of naïve mature B-cells in fully developed mice.

```r
# Visualize ConDecon's inferred cell abundance for bulk samples from 1 month
PlotConDecon(ConDecon_obj = ConDecon_obj,
             umap = scRNA_meta_data[,c("UMAP_1", "UMAP_2")],
             samples = row.names(BulkRNA_meta_data)[BulkRNA_meta_data$Age_months == 1],
             cells = row.names(scRNA_meta_data)[scRNA_meta_data$cellType %in%
                c("Pro_B_cells", "Immature_B_cells", "late pro-B cell", "Naive_B_cells",
                                                              "Precursor B cells")])
```

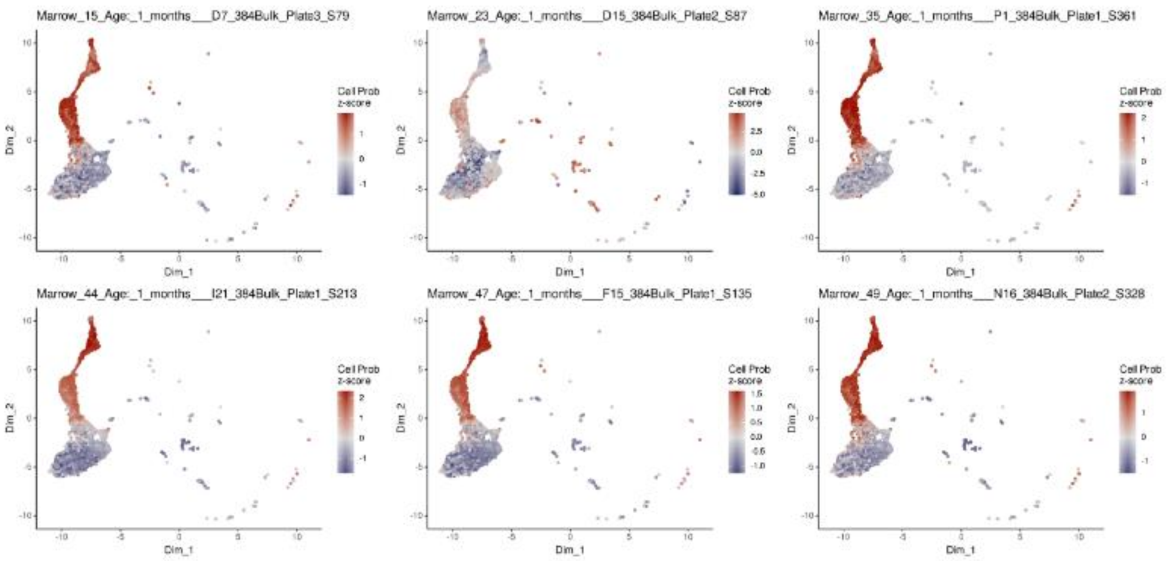

```r
# Visualize ConDecon's inferred cell abundance for bulk samples from 12 months
PlotConDecon(ConDecon_obj = ConDecon_obj,
             umap = scRNA_meta_data[,c("UMAP_1", "UMAP_2")],
             samples = row.names(BulkRNA_meta_data)[BulkRNA_meta_data$Age_months == 12],
             cells = row.names(scRNA_meta_data)[scRNA_meta_data$cellType %in%
                c("Pro_B_cells", "Immature_B_cells", "late pro-B cell", "Naive_B_cells",
                                                           "Precursor_B_cells")])
```

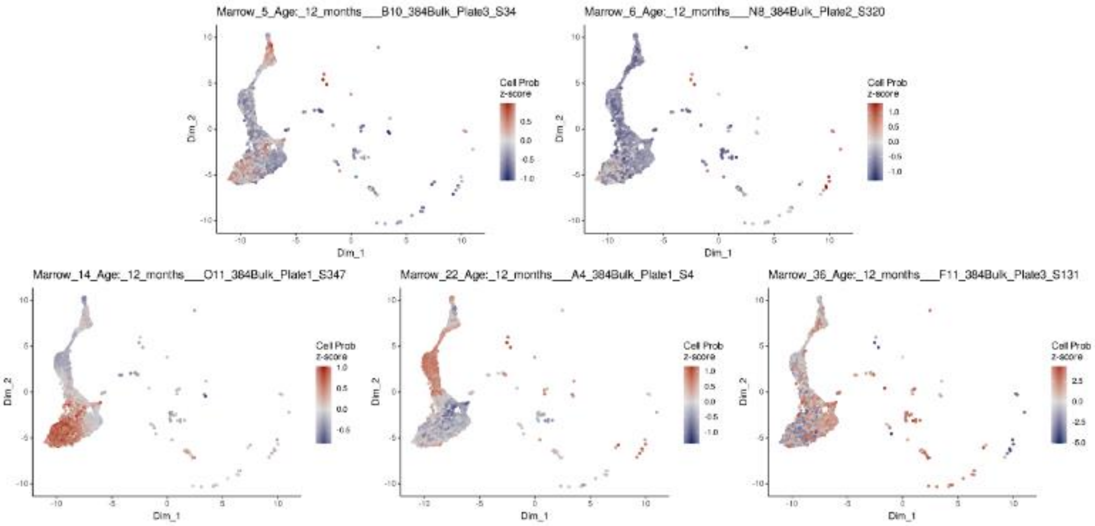


```r
# Visualize ConDecon's inferred cell abundance for bulk samples from 24 months
PlotConDecon(ConDecon_obj = ConDecon_obj,
             umap = scRNA_meta_data[,c("UMAP_1", "UMAP_2")],
             samples = row.names(BulkRNA_meta_data)[BulkRNA_meta_data$Age_months == 24],
             cells = row.names(scRNA_meta_data)[scRNA_meta_data$cellType %in%
                c("Pro_B_cells", "Immature_B_cells", "late pro-B cell", "Naive_B_cells",
                                                           "Precursor_B_cells")])
```

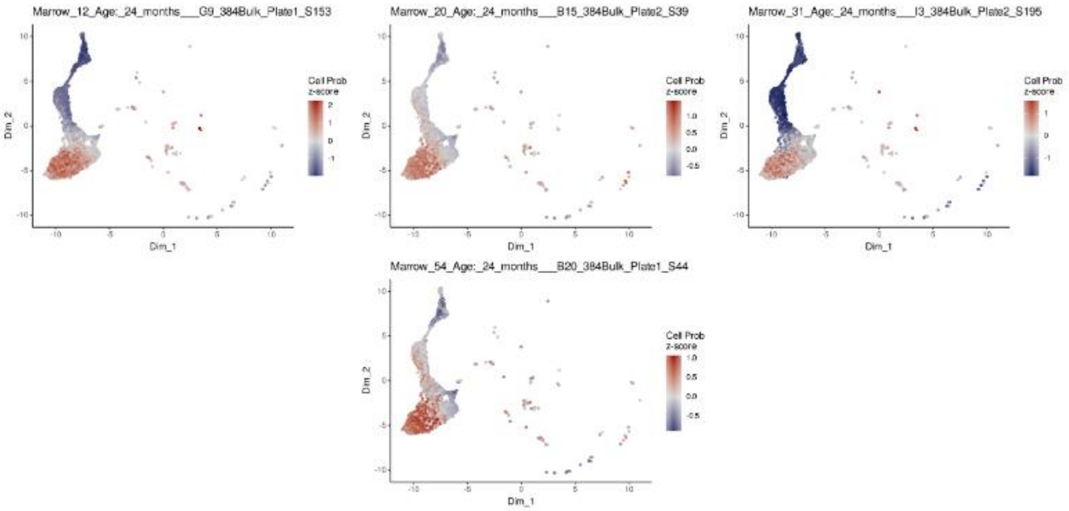

We can now use the cell probabilities inferred by ConDecon to deconvolve the pseudotime in bulk RNA-seq data. Using `TransferFeatures()`, we will estimate the pseudotime of B-cells within each bulk sample and compare these to the age of each mouse.

```
ConDecon_obj = TransferFeatures(ConDecon_obj = ConDecon_obj,
                                feature = scRNA_meta_data$monocle3_pseudotime)
# Transferring scRNA_meta_data$monocle3_pseudotime...
```

Here we will visualize the inferred B-cell pseudotime in the single-cell RNA-seq data.

```
# Visualize the pseudotime of the B cells in the single-cell RNA-seq data
ggplot(scRNA_meta_data, aes(x = UMAP_1, y = UMAP_2, color = monocle3_pseudotime)) +
  geom_point(size = 0.5) +
  scale_color_viridis_c(option = "plasma") +
  theme_classic()
```

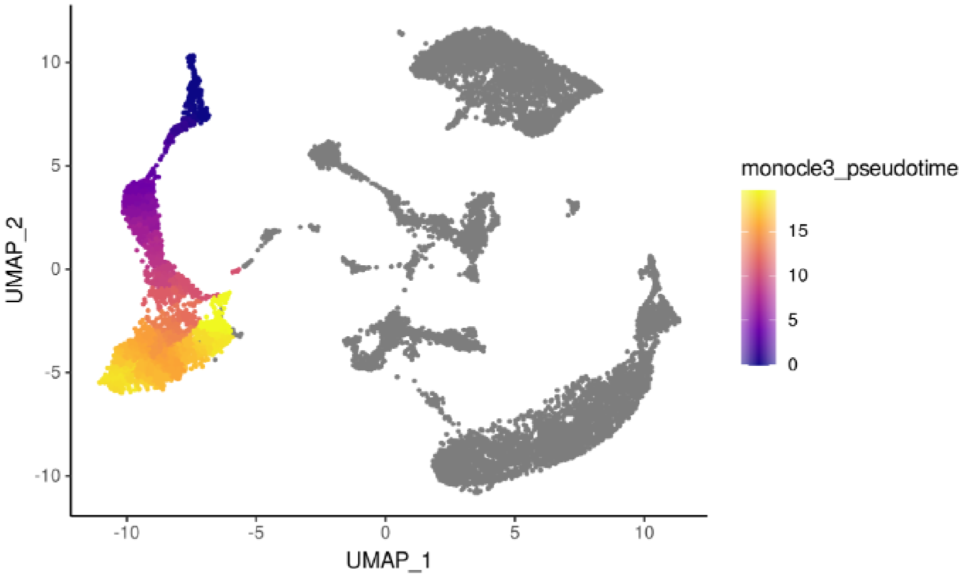

We can compare the estimated pseudotime of B-cells within each bulk sample to the age of each bulk sample. Consistent with the notion that there is a shift from an abundance of Pro B-cells in young mice to an abundance of Naive B-cells in fully developed mice, we find a strong correlation between ConDecon's estimated B-cell pseudotime and the age of the mouse in each bulk sample.

```
ggplot(data.frame(age_months = BulkRNA_meta_data$Age_months, predicted_B_cell_pseudo = ConDecon_obj$TransferFeatures[1,]),
       aes(x = age_months, y = predicted_B_cell_pseudo)) +
  geom_point(size = 2) +
  ggtitle(paste0("Pearson's correlation: ", round(cor(ConDecon_obj$TransferFeatures[1,],
                                                   as.numeric(BulkRNA_meta_data$Age_months)), 2))) +
  theme_classic()
```

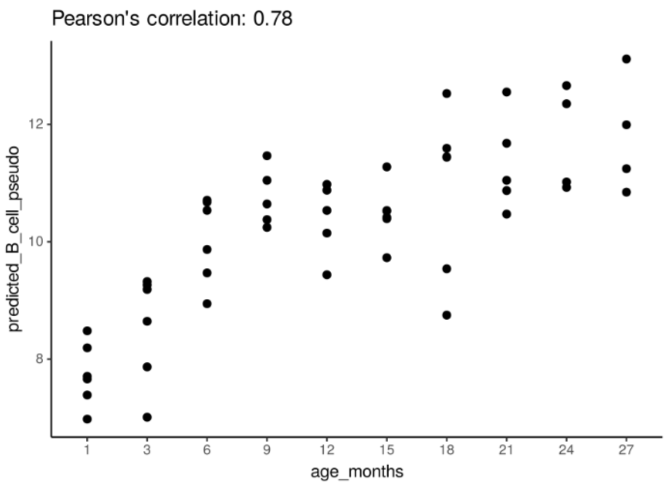

Deconvolution of spatial transcriptomic data

ConDecon's general approach for estimating cell abundances can be applied to other omics data modalities such as spatial transcriptomics. In this example, we will apply ConDecon to stereo-seq and single-cell RNA-seq data of 10 zebrafish embryos profiled 3.3 hours post-fertilization [S5]. For convenience, the data associated with this example is available on Zenodo (https://zenodo.org/record/7604026#.Y-EzEC_MIuU).

```
library(ConDecon)
library(ggplot2)
library(gridExtra)
```

As a reference dataset, we will use single-cell RNA-seq data of embryos 3.3 hpf from the same study and used diffusion pseudotime to parameterize the differentiation of blastomere cells in these data. We will start by loading in the single-cell RNA-seq count and meta data.

```r
scRNA_count <- readRDS("../scRNA_count.Rds")
scRNA_PCA <- readRDS("../scRNA_PCA.Rds")
scRNA_var_genes <- readRDS("../scRNA_var_genes.Rds")
scRNA_meta_data <- readRDS("../scRNA_meta_data.Rds")
```

```r
# Visualize the cell type annotations and predicted pseudotime
# of the single-cell RNA-seq data
p1 <- ggplot(scRNA_meta_data, aes(UMAP1, UMAP2, color = clusters)) +
  geom_point(size = 0.75) + theme_classic()
p2 <- ggplot(scRNA_meta_data, aes(UMAP1, UMAP2, color = dpt_pseudotime)) +
  geom_point(size = 0.75) + scale_color_viridis_c(option = "plasma") + theme_classic()
grid.arrange(p1, p2, ncol=2)
```

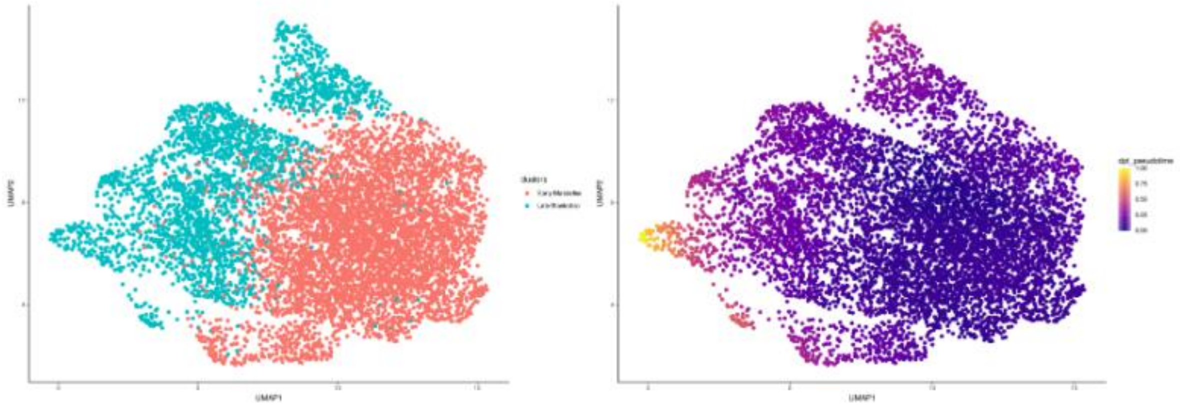

We are interested in using ConDecon to deconvolve each pixel of the tissue section profiled by stereo-seq. To reduce the computational time necessary to run this example, we have included the stereo-seq data associated with a one tissue section in Zenodo.

```r
stereoseq_slice1_RNA_data <- readRDS("../stereoseq_slice1_RNA_data.Rds")
stereoseq_meta_data <- readRDS("../stereoseq_meta_data.Rds")
```

```r
# Visualize the cell type annotations of the stereo-seq data from 1 tissue section
ggplot(stereoseq_meta_data, aes(spatial_x, spatial_y, color = bin_annotation)) +
  geom_point() +
  theme_linedraw()
```

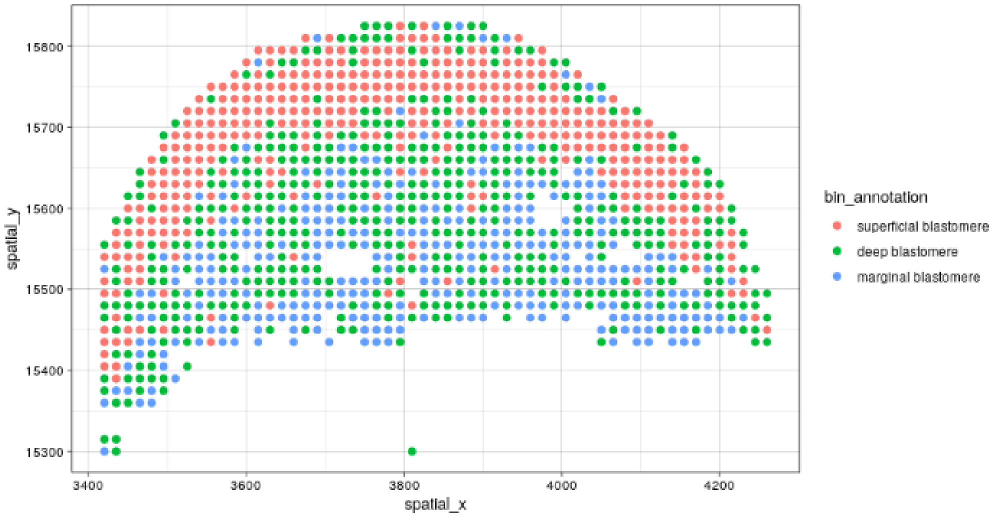

We can now use `RunConDecon()` to deconvolve this spot-based spatial transcriptomic data using single-cell RNA-seq data as reference.

```r
startTime <- Sys.time()

ConDecon_obj <- RunConDecon(counts = scRNA_count,
                            latent = scRNA_PCA,
                            variable.features = scRNA_var_genes,
                            bulk = stereoseq_slice1_RNA_data,
                            max.cent = 1)

endTime <- Sys.time()
print(endTime - startTime)
# Time difference of 4.49636 mins/Time difference of 6.747742 mins
```

With `PlotConDecon()`, we can visualize the relative cell probabilities of 3 example spots.

```
# Let's visualize the cell abundance distribution of 3 example spots
PlotConDecon(ConDecon_obj,
             umap = scRNA_meta_data[, c("UMAP1", "UMAP2")],
             samples = c("DNB_3795_15810", "DNB_3945_15525", "DNB_4080_15600"))
```

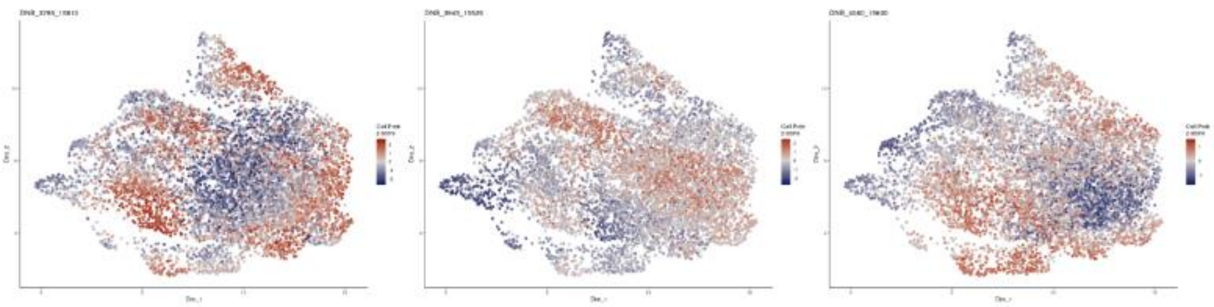

```
# Identify the location of these spots on the tissue section
ggplot(stereoseq_meta_data, aes(spatial_x, spatial_y, color = three_examples)) +
  geom_point() +
  scale_color_manual(values=c("#009E73", "#E69F00", "#56B4E9", "#CCCCCC")) +
  theme_linedraw()
```

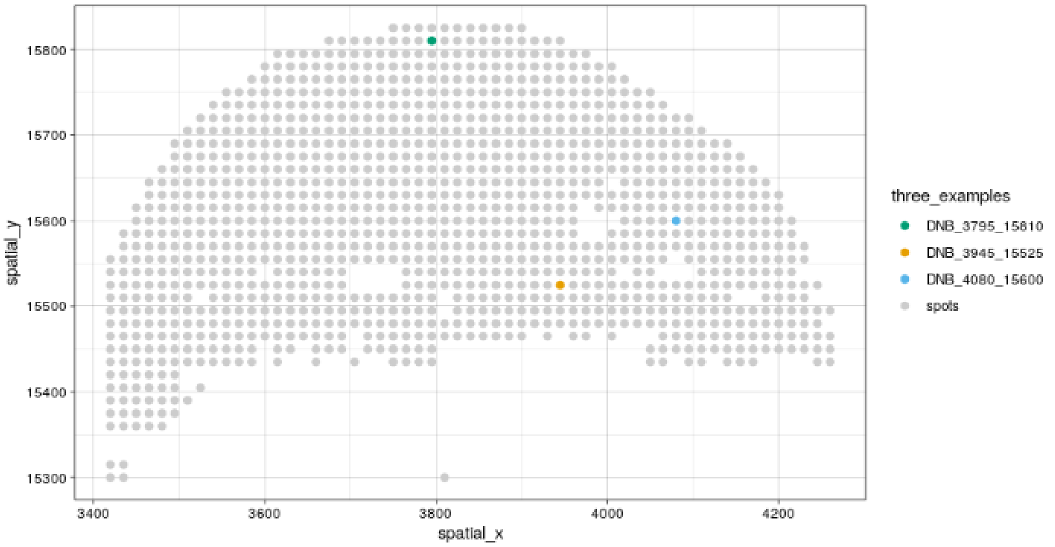

We can now use the cell probabilities inferred by ConDecon for each pixel to deconvolve pseudotime in the spatial data. Using `TransferFeatures()`, we will transfer the pseudotime estimates from the reference single-cell data to the stereo-seq data.

```
ConDecon_obj = TransferFeatures(ConDecon_obj = ConDecon_obj,
                                feature = scRNA_meta_data$dpt_pseudotime)
```

The resulting trajectories recapitulated the known spatial patterns of cell differentiation in the blastodisc, where the differentiation sequence progresses from marginal blastomere cells into deep and superficial blastomere cells.

```
# Visualize the pseudotime estimates for each pixel
ggplot(stereoseq_meta_data, aes(spatial_x, spatial_y, color = ConDecon_obj$TransferFeatures[1,])) +
  geom_point() +
  scale_color_viridis_c(option = "plasma") +
  theme_linedraw()
```

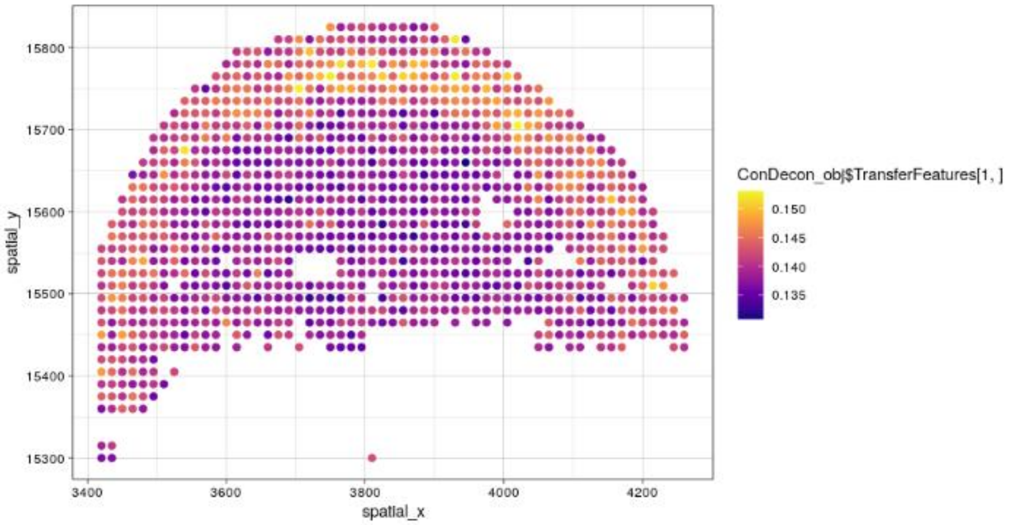


```
meta_data <- data.frame(celltype = stereoseq_meta_data$bin_annotation,
                        transfer_pstime = ConDecon_obj$TransferFeatures[1,])

ggplot(meta_data, aes(x=celltype, y=transfer_pstime, color = celltype)) +
  geom_violin() +
  geom_boxplot(width=0.3) +
  theme_classic()
```

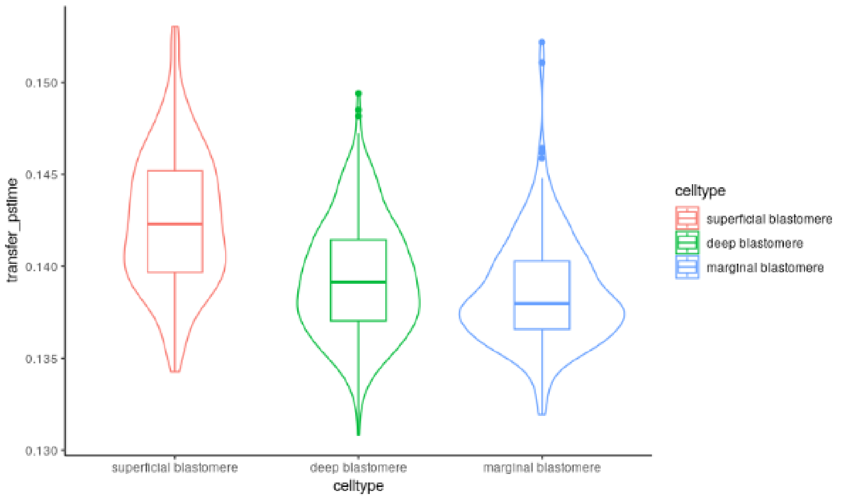

Deconvolution of chromatin accessibility data

In this example, we will apply ConDecon to bulk and single-cell ATAC-seq data from patient-derived melanoma cell cultures where the SOX10 transcription factor was knocked down by siRNA. Cells were then sampled for sequencing at 0, 24, 48, and 72 hours after SOX10 knockdown [S6]. For convenience, the processed data associated with this tutorial is available on Zenodo (https://zenodo.org/record/7604026#.Y-EzEC_MIuU).

To apply ConDecon to chromatin accessibility data, the bulk and single-cell ATAC-seq data must contain a common set of peaks. We created this by binning the genome into non-overlapping 10KB bins and aggregating the peaks from the single-cell and bulk ATAC-seq data into those bins.

```
library(ConDecon)
library(ggplot2)
```

We will use single-cell ATAC-seq data from each of the four sampling times as a reference dataset.

```
snATAC_count <- readRDS("../snATAC_count.Rds")
snATAC_SVD <- readRDS("../snATAC_SVD.Rds")
snATAC_var_genes <- readRDS("../snATAC_var_genes.Rds")
snATAC_meta_data <- readRDS("../snATAC_meta_data.Rds")
```

```
# Visualize the sampling time of the single-cell ATAC-seq data
ggplot(snATAC_meta_data, aes(x = UMAP_1, y = UMAP_2, color = hrs)) +
  geom_point(size = 3) +
  theme_classic()
```

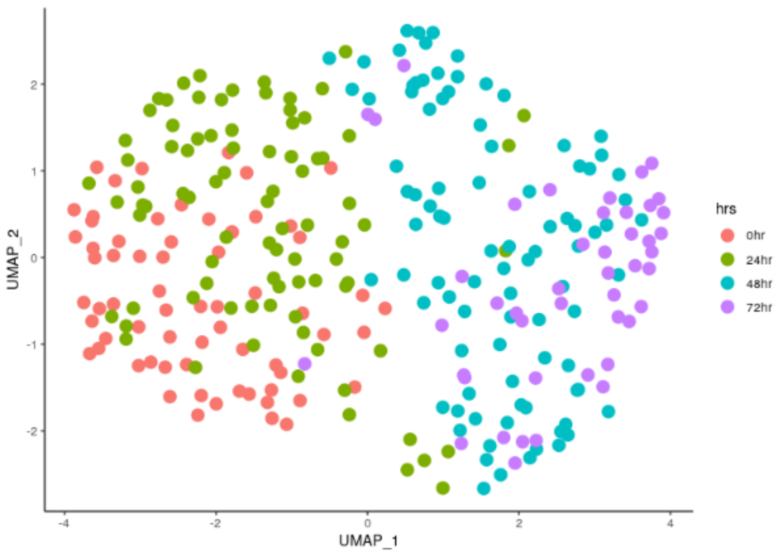

We are interested in using ConDecon to deconvolve 8 bulk ATAC-seq samples from 2 different patients (MM087 and MM057) and each of the four sampling times.

```
BulkATAC_data <- readRDS("../BulkATAC_data.Rds")
BulkATAC_meta_data <- readRDS("../BulkATAC_meta_data.Rds")
```

We can now use `RunConDecon()` to deconvolve bulk ATAC-seq data using single-cell ATAC-seq data as reference.

```
startTime <- Sys.time()

ConDecon_obj = RunConDecon(counts = snATAC_count,
                           latent = snATAC_SVD[,2:20],
                           bulk = BulkATAC_data,
                           variable.features = snATAC_var_genes)

endTime <- Sys.time()
print(endTime - startTime)
#Time difference of 1.620496 mins
```

With `PlotConDecon()`, we can visualize the relative cell probabilities of each bulk sample. We find that ConDecon infers a higher abundance of reference cells from the same sampling time as the query bulk sample, independently of the specific patient cell line of the query sample.

```
# Let's visualize the cell abundance distribution of all 8 bulk samples
PlotConDecon(ConDecon_obj = ConDecon_obj,
             umap = snATAC_meta_data[, c("UMAP_1", "UMAP_2")],
             pt.size = 3)
```

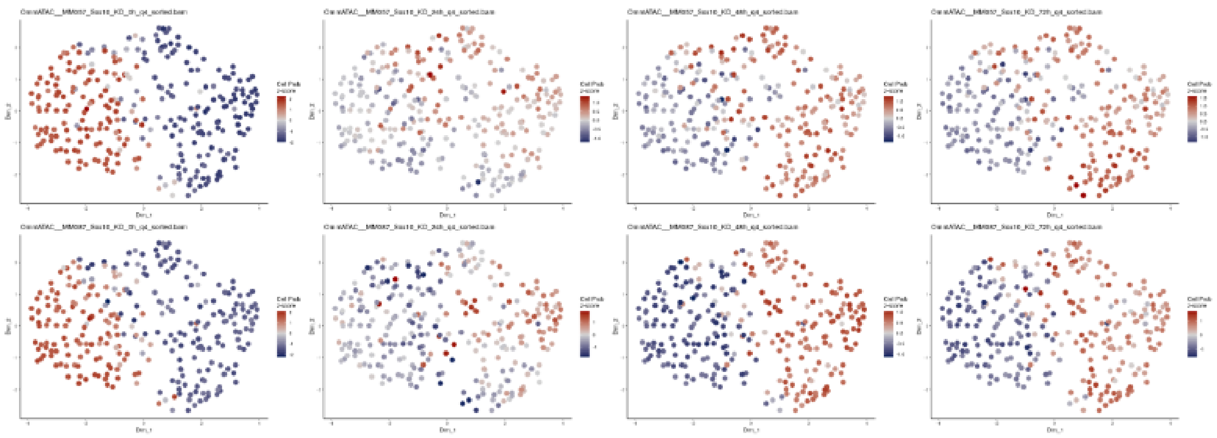

We can now use the cell probabilities inferred by ConDecon to deconvolve the sampling time of the bulk ATAC-seq data. Using `TransferFeatures()`, we will estimate the sampling time of each bulk sample and compare these to the actual sampling time.

```r
ConDecon_obj = TransferFeatures(ConDecon_obj = ConDecon_obj,
                                feature = snATAC_meta_data$time_hrs)
```

```r
meta_data <- data.frame(bulk_time = BulkATAC_meta_data$hrs,
                        transfer_feature = ConDecon_obj$TransferFeatures[1,],
                        patients = BulkATAC_meta_data$patient)

ggplot(meta_data, aes(x = bulk_time, y = transfer_feature, color = patients)) +
  geom_point(size = 3) +
  theme_classic()
```

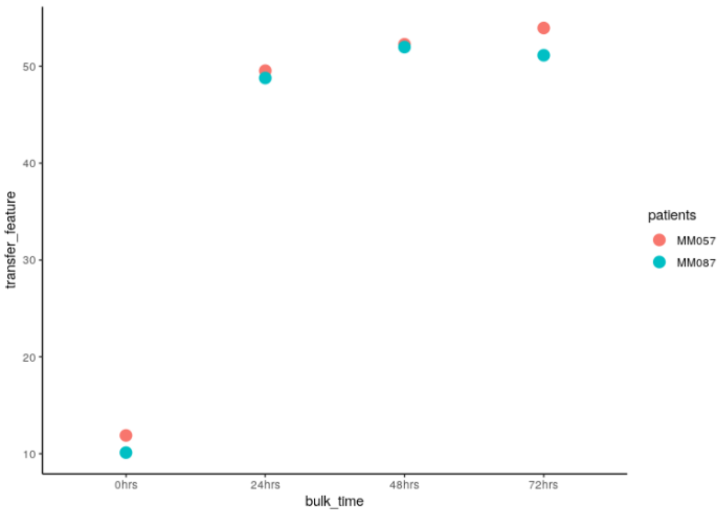

Supplemental References

[S1] Oetjen, K.A., Lindblad, K.E., Goswami, M., Gui, G., Dagur, P.K., Lai, C., Dillon, L.W., McCoy, J.P., and Hourigan, C.S. (2018). Human bone marrow assessment by single-cell RNA sequencing, mass cytometry, and flow cytometry. JCI Insight *3*. 10.1172/jci.insight.124928.

[S2] Hippen, A.A., Omran, D.K., Weber, L.M., Jung, E., Drapkin, R., Doherty, J.A., Hicks, S.C., and Greene, C.S. (2023). Performance of computational algorithms to deconvolve heterogeneous bulk ovarian tumor tissue depends on experimental factors. Genome biology *24*, 239. 10.1186/s13059-023-03077-7.

[S3] Newman, A.M., Steen, C.B., Liu, C.L., Gentles, A.J., Chaudhuri, A.A., Scherer, F., Khodadoust, M.S., Esfahani, M.S., Luca, B.A., Steiner, D., et al. (2019). Determining cell type abundance and expression from bulk tissues with digital cytometry. Nat Biotechnol *37*, 773-782. 10.1038/s41587-019-0114-2.

[S4] Tabula Muris Consortium. (2020). A single-cell transcriptomic atlas characterizes ageing tissues in the mouse. Nature *583*, 590-595. 10.1038/s41586-020-2496-1.

[S5] Liu, C., Li, R., Li, Y., Lin, X., Zhao, K., Liu, Q., Wang, S., Yang, X., Shi, X., Ma, Y., et al. (2022). Spatiotemporal mapping of gene expression landscapes and developmental trajectories during zebrafish embryogenesis. Dev Cell *57*, 1284-1298 e1285. 10.1016/j.devcel.2022.04.009.

[S6] Bravo Gonzalez-Blas, C., Minnoye, L., Papasokrati, D., Aibar, S., Hulselmans, G., Christiaens, V., Davie, K., Wouters, J., and Aerts, S. (2019). cisTopic: cis-regulatory topic modeling on single-cell ATAC-seq data. Nat Methods *16*, 397-400. 10.1038/s41592-019-0367-1.

[S7] Aubin, R.G., Troisi, E.C., Montelongo, J., Alghalith, A.N., Nasrallah, M.P., Santi, M., and Camara, P.G. (2022). Pro-inflammatory cytokines mediate the epithelial-to-mesenchymal-like transition of pediatric posterior fossa ependymoma. Nature communications *13*, 3936. 10.1038/s41467-022-31683-9.

[S8] Zappia, L., Phipson, B., and Oshlack, A. (2017). Splatter: simulation of single-cell RNA sequencing data. Genome biology *18*, 174. 10.1186/s13059-017-1305-0.
